# Amplicon sequences from enriched communities obtained at the mouth of tributaries along Lake Superior’s southern shore

**DOI:** 10.1128/mra.00235-25

**Published:** 2025-04-14

**Authors:** Fuad J. Shatara, Benjamin Davidson, Nimish Pujara, Erica L.-W. Majumder

**Affiliations:** 1 Department of Bacteriology, University of Wisconsin-Madison 205263, Madison, Wisconsin, USA; 2 Department of Civil and Environmental Engineering, University of Wisconsin-Madison 5228, Madison, Wisconsin, USA; Montana State University, Bozeman, Montana, USA

**Keywords:** Great Lakes, freshwater, microbial communities

## Abstract

Here, we present amplicon sequences from enriched communities of bulk and filtered water at 15 sites along Lake Superior’s southern shore, corresponding to the mouths of tributaries that feed into the Great Lake. Microbial communities forming along these sites provide insight into the effect of the tributaries on Lake Superior’s microbial communities.

## ANNOUNCEMENT

**Fig 1:**
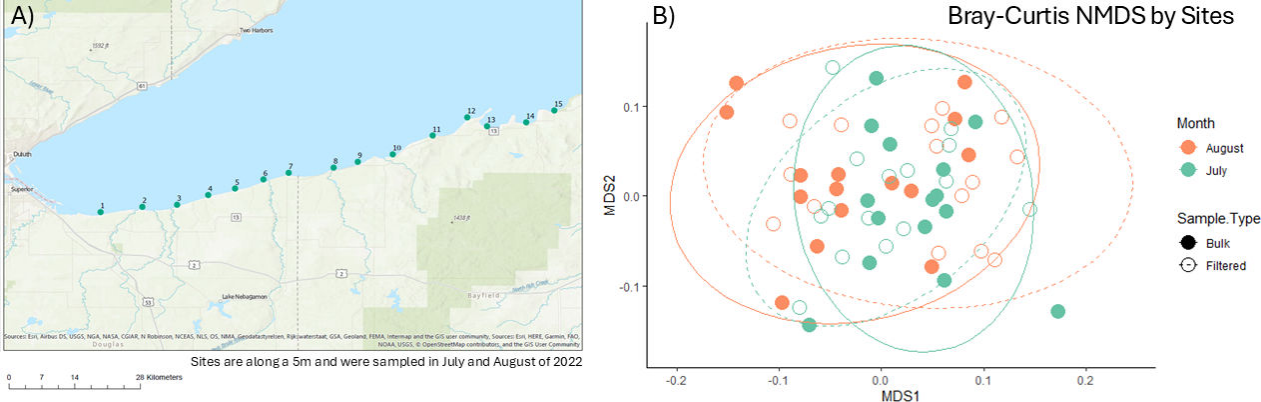
(A) Map of the 15 sample collection locations on the southern shore of Lake Superior where tributary rivers enter the lake. Bulk water and filtered water samples were collected from each of the 15 sites in both July and August 2022. Water quality parameters and other metadata are reported in reference 7. Reproduced with permission. (B) Microbial community beta diversity analyzed with a Bray-Curtis NMDS plot by sample site. Dashed ellipses represent microbial communities from filtered water samples, and solid ellipses represent bulk water samples.

Lake Superior’s southern shore experienced unexpected algal blooms within the last decade, despite a lack of confirmed blooms historically (1, 2). Anthropogenic runoff and climate change are suggested to be driving factors in the formation and proliferation of algal blooms globally (3, 4). By sequencing microbial communities from the mouth of tributaries as they enter Lake Superior, we can gain a better understanding of how the runoff and debris within is impacting the overall microbial community structure (5, 6). During two sampling events in July and August 2022, from each of 15 sites (Fig. 1A map, metadata (7)), bulk water was collected from the surface of the lake along with water filtered through a 100 µm stainless-steel mesh screen in order to remove larger particulate matter (8).

Following the collection of the samples, 50 mL of bulk or filtered lake water was added to a flask containing 50 mL of BG-11 (9) media and cultured at room temperature with a 12 hour light-dark cycle under Life-GLO T-8 40W bulbs at a distance of 30 cm. After 30 days, 1.5 mL of each enriched culture was collected for 16S rRNA amplicon sequencing of the resulting cyanobacterial-associated community. DNA was extracted using the Qiagen DNeasy Power Water kit (Qiagen #14900-100-NF), and DNA concentrations were quantified using Qubit 3 Fluorometer (ThermoFisher Scientific #Q33216). For library preparation, DNA extracts were PCR amplified using dual-indexed primers targeting the V4 region of the 16S rRNA gene, designed with appropriate Illumina adapters according to Kozich et al. (10). PCR-amplified products were normalized using a SequalPrep Normalization kit (ThermoFisher Scientific A1051001) before being pooled for sequencing on an Illumina Miseq version 2. The resulting sequences were assembled and aligned using the Silva version 138.1 database (11, 12) and quality controlled by removing sequences with ambiguous base pairs, sequences outside the anticipated 200–500 bp length, sequences that did not align to the appropriate region, or sequences that were detected to be chimeras using UCHIME2 (13).

Following quality control, a total of 925,380 reads of the original 1,156,889 reads were retained (80%), containing 13,237 unique reads, with an average of 15,955 reads per sample across 58 samples. Bulk water samples contained an average of 19,374 reads per sample, while filtered water samples contained an average of 19,188 reads per sample. GC content ranged from 48% to 55% across all samples, and a total of 4,232 operational taxonomic units (OTUs) were identified at a 97% similarity threshold, with bulk water samples averaging 218 OTUs per sample and filtered water samples averaging 202. Across all samples, coverage ranged from 97.3% to 99.9% with an average coverage of 99.3%. Among the communities, the average Shannon diversity index for filtered water communities was 3.02, while the bulk water average was 2.83. Non-metric multidimensional scaling of the Bray-Curtis dissimilarities among bulk and filtered water communities was used to visualize distinctions between these communities (Fig. 1B). In summary, this data set allows for the identification of the influence of tributaries into Lake Superior’s southern shore, with the potential to aid in characterizing the influence of these rivers on algal bloom formation.

## Data Availability

Sequencing reads and sampling metadata, such as location coordinates, are publicly available on the NCBI sequencing read archive under BioProject accession PRJNA1216635. The Wisconsin Department of Natural Resources report EGAD: 3900-2024-02 contains additional metadata for each site and samples, including water quality parameters. This report can be found through the Electronic Guidance and Documents database at https://apps.dnr.wi.gov/water/egadSearch.aspx using report number EGAD 3900-2024-02.
